# Influence of Vitamin A supplementation on inflammatory biomarkers in adults: a systematic review and meta-analysis of randomized clinical trials

**DOI:** 10.1038/s41598-022-23919-x

**Published:** 2022-12-10

**Authors:** Mohammad Gholizadeh, Poorya Basafa Roodi, Faezeh Abaj, Sakineh Shab-Bidar, Ahmad Saedisomeolia, Omid Asbaghi, Mahshid lak

**Affiliations:** 1 Department of Clinical Nutrition and Dietetics, Faculty of Nutrition and Food Technology, Shahid Beheshti University of Medical Sciences, Tehran, Iran; 2 Department of Nutrition, School of Public Health, Iran University of Medical Sciences, Tehran, Iran; 3 Department of Community Nutrition, School of Nutritional Sciences and Dietetics, Tehran University of Medical Sciences, Tehran, Iran; 4 School of Human Nutrition, Research Associate, McGill University, 21,111 Lakeshore, Ste-Anne-de-Bellevue, Quebec, H9X 3V9 Canada; 5 Cancer Research Center, Shahid Beheshti University of Medical Sciences, Tehran, Iran; 6 Department of Public Health, Faculty of Medical Sciences, Islamic Azad University of Arak, Arak, Iran

**Keywords:** Biomarkers, Molecular medicine

## Abstract

Vitamin A is an anti-oxidant which has been presumed to act as an anti-infective vitamin in many studies. This study aimed to evaluate the association between vitamin A supplementation and c-reactive protein (CRP), tumor necrosis factor-alpha (TNF-α), and interleukin 6 (IL-6) levels in randomized control trials (RCTs) studies on adults. A systematic search was performed on databases including PUBMED, SCOPUS, and the Cochrane library. The studies included were considered for data extraction and subsequently assessed for effect. Weighted mean differences (WMD) and 95% confidence intervals (CIs) were evaluated. Among 13,219 articles 13 studies were included for analysis of CRP and TNF-α, as well as 9 studies included for IL-6 in quality and quantity. The pooled WMD analysis of CRP demonstrated that vitamin A supplementation significantly increased CRP concentration with (WMD: 0.84 mg/L; 95% CI 0.29–1.39, I^2^ = 0.96.2% and p value < 0.003). However, there was no significant correlation between vitamin A supplementation and lower plasma TNF-α (p < 0.45)). Subgroup analysis by dosage demonstrate significant association between vitamin A supplementation and IL-6 in dosage with 50,000 with (WMD: − 1.53 mg/L; 95% CI − 2.36 to − 0.71, p value < 0.00001) as well as a negative significant association was seen at 44 weeks of supplementation with 50,000 IU/day retinyl palmitate and TNF-a in chronic hepatitis B conditions with (− 0.94 (− 1.19, − 0.69) p < 0.0001). The result of this study demonstrates that supplementation of vitamin A at low and high dosages for short and long durations increases the CRP plasma concentrations on adults and vitamin A supplementation decreases the TNF-α concentrations in chronic hepatitis B on adults. Therefore, there is an inverse association between vitamin A supplementation and plasma and fecal IL-6 concentrations in many infection conditions.

## Introduction

Inflammation is characterized by increased cytokines in the blood flow during tissue injury^1^. Acute phase cytokines are increased during the inflammatory process. These cytokines include interleukin-6^2^ (IL-6), interleukin-1 (IL-1), high sensitivity C-reactive protein (hs-CRP), and tumor necrosis factor-alpha (TNF-α)^3–6^. hs-CRP predicts the development of cardiovascular diseases (CVD), diabetic mellitus^7^, hypertension (HTN), metabolic syndrome (Mets), obesity, and cancer^4,8,9^. IL-6 is an initiator of inflammation in the acute phase and induces the synthesis of other acute-phase inflammatory biomarkers. Together, IL-6 and hs-CRP are the best indicators for assessing inflammation and the risks associated with acute inflammation^10^. IL-6 has also been shown to be an important biomarker for assessing the severity of coronavirus outcomes^11^. Furthermore, reactive oxygen species (ROS) are increased during inflammatory conditions. ROS are removed from the body by antioxidant compounds. Imbalances between ROS and antioxidants cause progressive inflammation^12,13^. Important antioxidants for decreasing ROS include ascorbic acid and vitamin E^14^. Additionally, vitamin A is an antioxidant compound that plays an important beneficial role to combat oxidation and inflammation^15^.

Vitamin A has been shown to be an anti-infective fat soluble vitamin playing an important beneficial role in common infectious diseases^16,17^. Vitamin A deficiency leads to the suppression of the immune system and increases the rate of mortality among children^18–22^. Retinol is a natural metabolite of vitamin A, and plasma levels of retinol indicate vitamin A status^23,24^. Retinol levels are reduced during acute inflammation through an increased urinary retinol excretion; a decreased gastrointestinal retinol absorption; and a lowered synthesis of retinol-binding protein (RBP) by the liver^25–28^.

The human body needs vitamin A, which is a fat-soluble vitamin. Based on large studies, adult men should consume 900 µg of vitamin A per day^29^, while adult women should consume 700 µg^30^. Two major kinds of vitamin A, bioactive forms of vitamin A (retinol and retinyl ester) and provitamin A (carotenoids) are both accessible in the human diet^30–32^. Animal-based foods, such as fish, meat, and dairy products contain preformed vitamin A. Also, other forms of vitamin A like carotenoid, which comprises alpha and beta carotene can be transformed into retinol in the human body, is a type of vitamin A found in yellow, orange, and red fruits and vegetables^30–33^.

Previous studies have recommended high doses of vitamin A in children suffering from diarrhea and measles^34^. Furthermore, vitamin A deficiency predicts the risk of tuberculosis, which can be ameliorated by vitamin A supplementation^35^. In animal models, vitamin A deficiency is accompanied by increased levels of inflammatory cytokines, including interferon-gamma, IL-6^2^, and IL-13^22^. Also, vitamin A deficiency has been seen to impair Immunoglobulin A (IgA) immune function in respiratory virus vaccines^36^. Based on the current literature, vitamin A is presumed to act as an anti-infection vitamin, boosting the body against infections; thus, it plays an important role to decrease inflammatory biomarkers^37,38^.

So far, to the best of the authors knowledge, there has been no systematic review or meta-analysis determining the influence vitamin A supplementation and levels of inflammatory biomarkers. Therefore, the aim of this study is to analyze the supplementation of vitamin A and levels of the inflammatory biomarkers including IL-6, TNF-alpha and CRP in adults.

## Methods

This study was performed and reported in accordance with the Preferred Items for Systematic Reviews and Meta Analyses (PRISMA) statement guidelines^39^.

### Search strategy

This study also describes PICO (population (adults), intervention (vitamin A), control (Control or comparison intervention), and outcomes (inflammatory biomarkers)) criteria in framing a research question. A literature search was performed on SCOPUS, PUBMED, and Cochrane databases to identify eligible studies. The search method was carried out based on the following keywords: "Vitamin A"[Title/Abstract] OR "Retinyl palmitate"[Title/Abstract] OR "retinoic acid"[Title/Abstract] OR "Retinol"[Title/Abstract] OR "All-Trans-Retinol"[Title/Abstract] OR "All Trans Retinol"[Title/Abstract] OR "Vitamin A1"[Title/Abstract] OR "11-Cis-Retinol"[Title/Abstract]) AND ("C-reactive protein"[Title/Abstract] OR "C reactive protein "[Title/Abstract] OR "CRP"[Title/Abstract] OR "High Sensitivity C-Reactive Protein"[Title/Abstract] OR "hs-CRP"[Title/Abstract] OR "hsCRP"[Title/Abstract]) AND "tumor necrosis factor alpha"[Title/Abstract] OR "TNF-αlpha"[Title/Abstract] OR "TNF-α"[Title/Abstract] AND "IL-6"[Title/Abstract] OR "interleukin 6"[Title/Abstract] OR "interleukin-6"[Title/Abstract]). Search mesh terms were carried out in both title and abstract. We manually searched Google Scholar as well as the references of the included publications to make sure no research was left out. The final search update was carried out on 28 September 2022.

### Inclusion and exclusion criteria

In our study, we considered trials that met the following criteria: (1) randomized controlled trials, (2) studies that have placebo groups, (3) consisted of use of vitamin A supplementation, (4) RCTs with at least 1-week duration of intervention, (5) studies that assessed inflammatory biomarkers as an outcome for both intervention and control group. (6) studies in English language.

Studies with the following criteria were excluded from the study: (1) studies not meeting the necessary inclusion criteria; (2) studies on animals, in vitro models, editorial papers, secondary studies, cohort settings, cross-sectional studies, case report studies, communication report; (3) duplicated studies; and (4) studies without supplementation placebo in the control group (randomized cluster trails) (5) short term studies (< 1 weeks).

### Data extraction

The following data were extracted: first author, year of publication, country of origin, participant numbers, mean age, gender, participants’ baseline health status type and dose of vitamin A supplementation, and the duration of the intervention. The data initially import into Excel (Microsoft Office, version 2016). Two authors independently [F.A] carried out the data extraction. Additionally, following data extraction, duplicate articles were eliminated, and article titles and abstracts were taken into account for inclusion and exclusion.

### Statistical analysis

The mean and standard deviation of outcomes including CRP, TNF-α, and IL-6 in treatment and placebo groups were extracted. Then, the analysis was calculated by weighted mean differences (WMDs) between treatment and placebo groups. when mean changes were not reported, we calculated them by using this formula: mean change = final values − baseline values, and SD changes were calculated by following formula^40^:

$$\mathrm{SD change}=\sqrt{[(\mathrm{SD baseline})^2 + (\mathrm{SD final})^2 - (2\mathrm{R }\times \mathrm{ SD baseline }\times \mathrm{ SD final})}$$

In crossover studies, we calculated the mean and SD by combining the mean and SD changes in each arm (interventions or controls).

The heterogeneity between studies was evaluated in three following categories: I^2^ lower than 50% is considered as low heterogeneity; I^2^ higher than 50% was considered as moderate and high heterogeneity (> 75%), respectively^41^. The random effects model was used to know in case of existing high heterogeneity between the included studies. The publication bias to report of the studies was assessed by funnel plot and Egger test^42^. The sensitivity analysis was carried out for sensitivity to our conclusion. Also, subgroup analysis was performed for finding the source of heterogeneity. All statistical analyses were done using STATA, version 14.2 (Stata Corporation, College Station, TX, USA). p value less than 0.05 was considered significant. Also, the risk of bias RoB 2:0 performed for assessing risk of biases on studies.

### Certainty assessment

The overall certainty of evidence across the studies was graded according to the guidelines of the GRADE (Grading of Recommendations Assessment, Development, and Evaluation) Working Group. The quality of evidence could be classified into four categories according to the corresponding evaluation criteria: high, moderate, low, and very low^43^.

## Results

### Study selection

**Figure 1:**
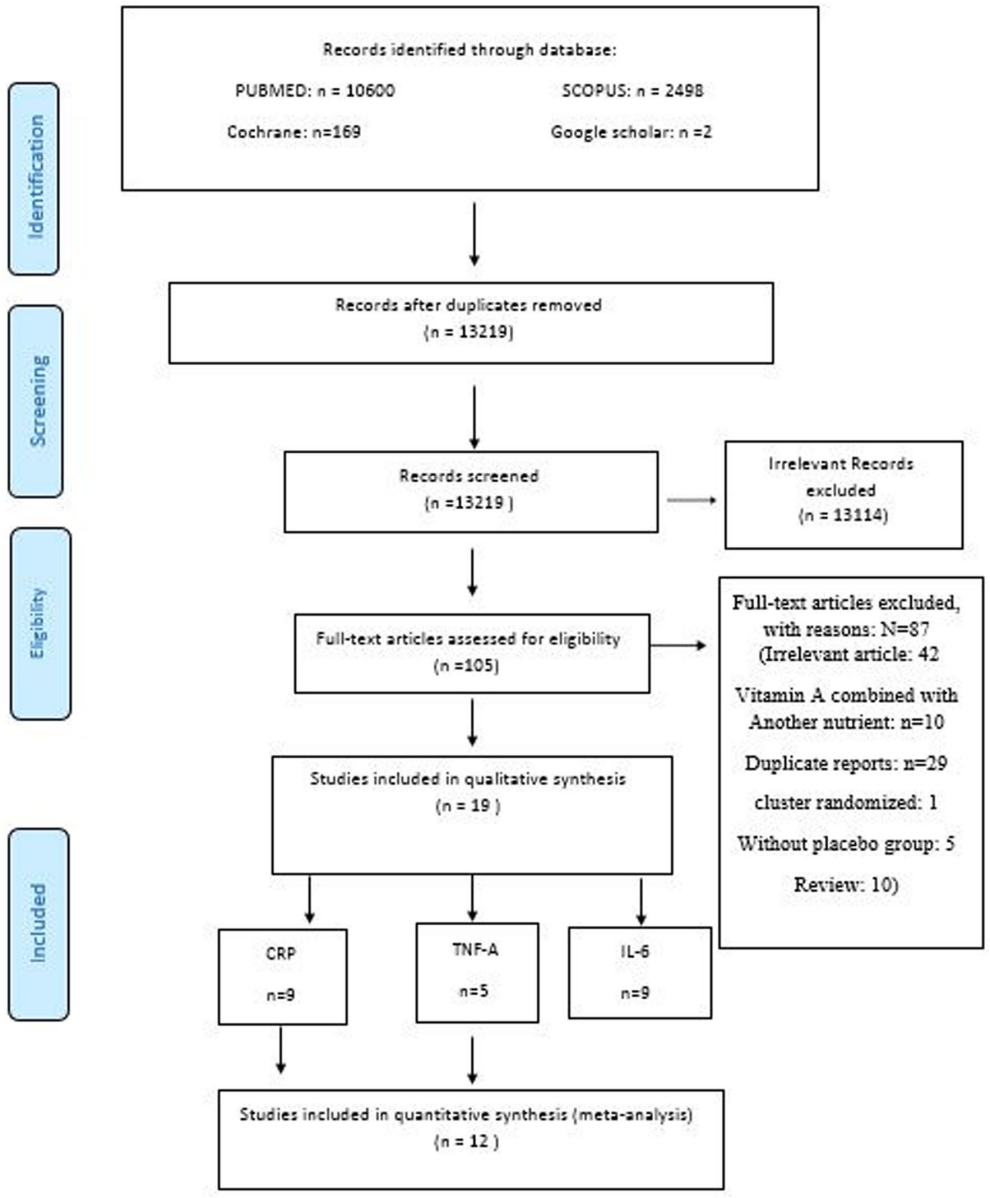
PRISMA chart of included studies for CRP, TNF-α, and IL-6.

A total of 13,269 studies were extracted from PUBMED, SCOPUS, Cochrane, and Google Scholars. After excluding the duplicated studies, 13,219 studies were considered for further screening. After reading the title/abstract and full text of studies based on the inclusion criteria, 19 studies were included for qualitative analysis. Additionally, 12 studies were included for quantitative analysis. The PRISMA flow diagram of the included studies is shown in Fig. 1.

### Study characteristics

The included studies (19 studies) were published from 1992 up to 2020. Of 19 studies, 12 were considered for quantitative analysis. In total, nine studies were included for CRP^2,44–51^, five studies for TNF-α analysis^2,44,52–54^. Nine studies that were ineligible for other analyses were included for IL-6^2,52,55–59^, seven studies were performed in Asia^2,21,44,46,52,60^, four studies in America^48,49,61^, and one study in Europe^53^. Except for two studies^46,62^, all studies included both male and female participants.

In total, the data for 2120, 221, and 482 individuals were available for C-reactive protein, TNF-α, and IL-6, respectively. The conditions of the study participants were tuberculosis (TB), measles related to pneumonia, sepsis, children with infections, worm infestation, pneumonia, low plasma retinol, and obesity. The duration of studies ranged from 2 weeks up to 24 weeks. All studies included for analysis administered vitamin A orally.

Two studies that administered vitamin A intravenously were excluded from the analysis. All studies reported the mean ± SD of the CRP and TNF-α in both treatment and placebo groups. The characteristics of the studies are shown in Table 1 for CRP and Table 2 for TNF-α. Additionally, the characteristics of studies assessing IL-6 are shown in Table 3.

**Table 1:** The characteristics of included studies for analysis.

Author, year	Country	Mean age	Sample size	Dose (mg)	Duration of treatment (weeks)	Health condition	Intervention type	Sex	CRP change in intervention and placebo group ^a^
Mahdieh Abbasalizad Farhangi (2013)	Iran	36	50	25,000	16	Obese	Retinyl palmitate	F	6.24 vs, 5.51
Shaikh M Ahmad (2020)	Bangladesh	0.5	271	50,000	6	Infants	Retinol	F/M	0.38 vs, 0.41
S. A. Tanumihardjo (2020)	USA	6	93	1333	12	Low serum retinol	Retinol	F/M	0.58 vs, 0.75
Charles B Stephensen (2002)	USA	5	92	200,000	2	Pneumonia	Retinol	F/M	30 vs, 38.6
Francisco J. Rosales (2002) (a)	USA	8.5	88	200,000	2	Pneumonia	Retinol	F/M	22.03 vs, 19.37
Francisco J. Rosales (2002) (b)	USA	8.5	108	200,000	2	Pneumonia	Retinol	F/M	17.83 vs, 16.28
Trevino A Pakasi (2010) (a)	Indonesia	35	136	5000	8	TB	Retinol	F/M	4 vs, 6.4
Trevino A Pakasi (2010) (b)	Indonesia	35	136	5000	8	TB	Retinol	F/M	1.5 vs, 1.7
Trevino A Pakasi (2010) (c)	Indonesia	35	119	5000	8	TB	Retinol	F/M	4.2 vs, 6.4
Trevino A Pakasi (2010) (d)	Indonesia	35	119	5000	8	TB	Retinol	F/M	1.1 vs, 1.3
Shaikh M Ahmad (2008)	Bangladesh	23.9	36	200,000	1	Low serum retinol	Retinol	M	1.27 vs, 0.75
Ph Donnen (a) (2001)	Brussels	3	542	200,000	1	During infection	Retinyl palmitate	F/M	34.6 vs, 31.9
Ph Donnen (b) (2001)	Brussels	3	540	5000	1	During infection	Retinyl palmitate	F/M	32.2 vs, 31.9
Sima Jafarirad (2013)	Iran	32.5	35	200,000	24	MS	Retinyl palmitate	F/M	2.88 vs, 1.58

Summary of clinical trials on the effects of vitamin A supplementation on inflammatory biomarker (CRP). *CRP* C-reactive protein, *TB* tuberculosis, *M* male, *F* female, a Changes in cytokine concentrations are presented by common units for CRP (mg/L).

**Table 2:** The characterized of studies that included TNF-α analysis.

Author, year	Country	Sample size	Mean age	Sex	Dose (mg)	Duration of treatment (weeks)	Type	TNF-α change in intervention and placebo group ^a^
S. E. Cox (2006)	England	99	21	F	10,000	15	Vit A	0.24 vs. − 0.18
Mahdieh Abbasalizad Farhangi (2013)	Iran	50	38	F	25,000	16	Retinyl palmitate	− 0.02 vs. − 0.01
Shaikh M. Ahmad (2009)	Bangladesh	36	25	M	240,000	56	Vit A	− 0.2 vs. − 3
Sama Bitarafan (2019)	Iran	79	35	M/F	25,000	24	Retinyl palmitate	61.17 vs 110.59
Tingting Cai (2019)	Bangladesh	30	54	M	50,000	44	Vit A	− 4.17 vs. − 3.23

Summary of clinical trials on the effects of vitamin A supplementation on inflammatory biomarker (TNF-α). *TNF-α* tumour necrosis factor-α, *M* male, *F* female, a Changes in cytokine concentrations are presented by common units for TNF-α (pg/mL).

**Table 3:** The characterized of studies that included IL-6 analysis.

Author, year	Country	Sample size	Mean age	Sex	Dose (mg)	Duration of treatment (weeks)	Type	IL-6 change in intervention and placebo group
Shaikh M. Ahmad (2020)	England	36	25	F	10,000	56	Vit A	− 53.4 vs. 6.6
sama Bitarafan (2019)	Iran	36	35	F	25,000	180	Retinyl palmitate	8.86 vs. 5.03
Tingting Cai (2019)	Bangladesh	30	54	M	50,000	44	Vit A	− 3.23 vs. − 3.12

Summary of clinical trials on the effects of vitamin A supplementation on inflammatory biomarker (IL-6). *IL-6* interleukin 6, *M* male, *F* female, a changes in cytokine concentrations are presented by common units for IL-6 (pg/mL).

To evaluate the effect of vitamin A on CRP, firstly, the data in treatment and placebo groups were pooled. The random effect analysis was performed for finding heterogeneity in data. This model represented high heterogeneity between studies).

### The effects of vitamin supplementation on CRP, TNF-a and IL-6

**Figure 2:**
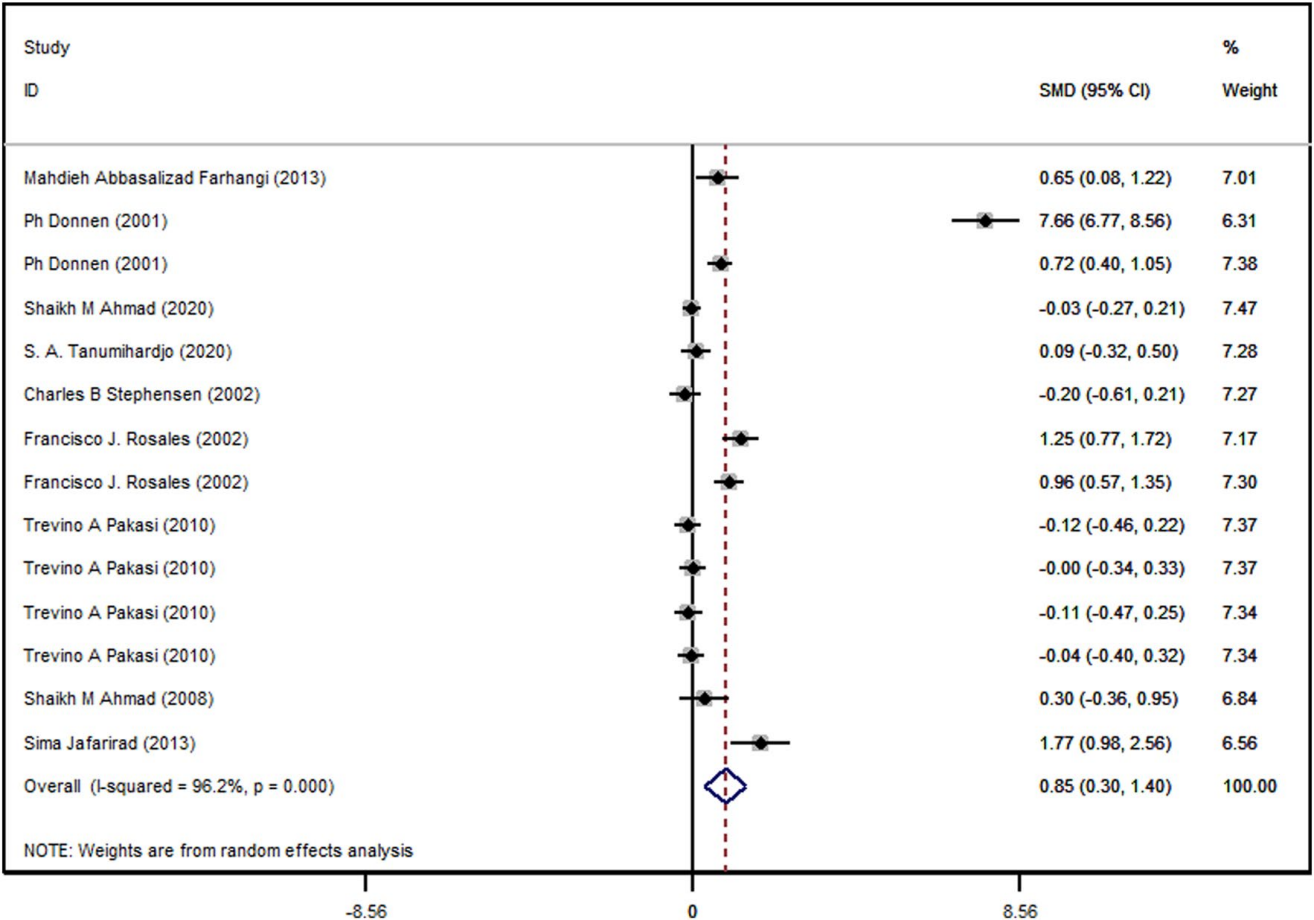
Forest plot for the effect of vitamin A supplementation on serum CRP concentrations, expressed as mean differences between intervention and control groups. Horizontal lines represent 95% CIs. *CRP* C-reactive protein, *SMD* standardized mean difference, *CI* confidence interval.

The calculated WMD of CRP was 0.84 mg/L (95% CI 0.29–1.39 I^2^ = 0.96.2% and p value < 0.003) (Fig. 2). There was no significant association between vitamin A and lower levels of CRP in the intervention group in comparison to the placebo group. A positive relationship was observed between vitamin A supplementation and CRP plasma concentration.

Sensitivity analysis was performed for assessing the potential effect of some studies in the result. We found no discernible robustness in the reporting of results across studies. Moreover, there was high heterogeneity between studies; subgroup analysis was performed to find these sources of heterogeneity.

When studies were sub-grouped by the duration of supplementation, the following categories were applied: supplementation with vitamin A for 1, 2, 6, 8, 12, 16, and 24 weeks. We did not observe a significant association in subgroup analysis by duration between studies.

When studies subgroup by intervention dosages there was a positively significant association observed at the supplementation dose of 25,000 IU/day (WMD: 0.65, 95% CI 0.07–1.2, heterogeneity = 00%, p value = 0.02).

However, subgroup analysis by conditions demonstrated a positive relationship in obese women measles-related pneumonia, multiple sclerosis (WMD: 0.65, 95% CI 0.08–1.2, p value = 0.02, WMD: 1.07, 95% CI 0.77–1.38, p value < 0.0001, WMD: 1.77, 95% CI 0.98–2.56, p value < 0.00001).

Also, subgroup by age showed significant positive associations in age groups 2–10 years old (p value < 0.004). Moreover, subgroup by sex showed significant positive associations in women and both (men and women, together) (p = 0.02), (p = 0.004), respectively.

**Figure 3:**
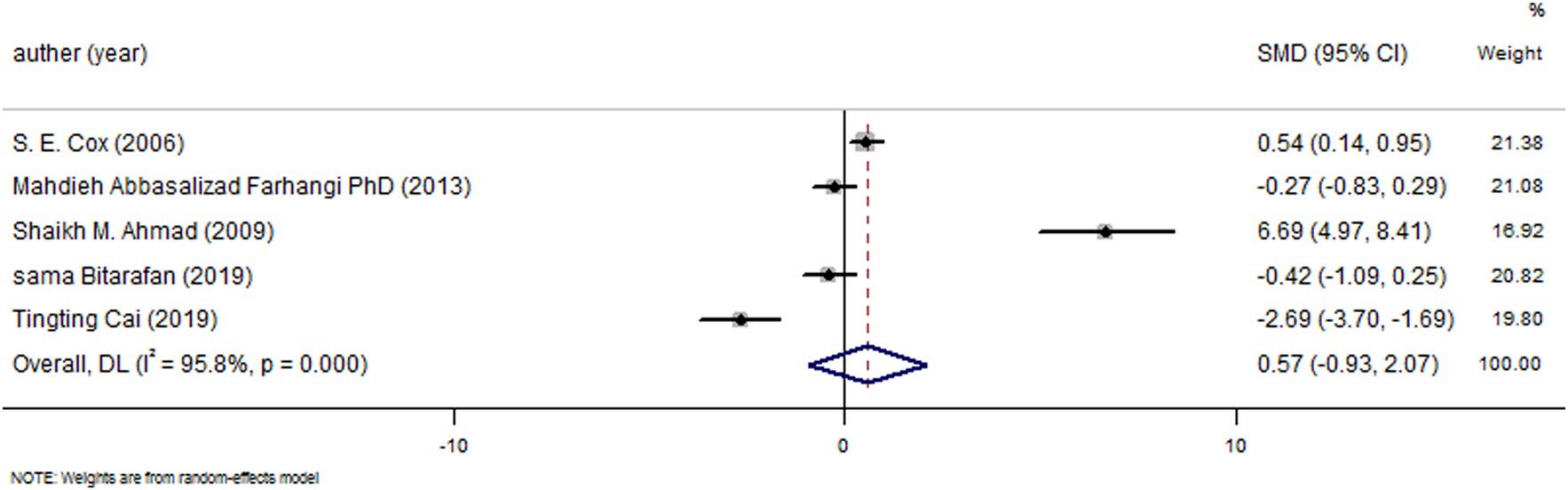
Forest plot for the effect of vitamin A supplementation on serum TNF-α concentrations, expressed as mean differences between intervention and control groups. Horizontal lines represent 95% CIs. *TNF-α* tumor necrosis factor-α, *SMD* standardized mean difference, *CI* confidence interval.

To assess the association between vitamin A supplementation and plasma TNF-α levels, pooled analysis data was carried out with mean ± SD in treatment and placebo groups. There was no significant correlation between vitamin A supplementation and lower plasma TNF-α (p < 0.45) (Fig. 3).

Based on duration subgroup analysis, a negative significant association was seen at 44 weeks of supplementation with 50,000 IU/day retinyl palmitate in chronic hepatitis B conditions with (− 0.94 (− 1.19, − 0.69) p < 0.0001), in contrast, positive association demonstrate at 15 and 56 weeks with 10.000 (0.42(0.11, 0.72) p < 0.0001) and 240,000 IU/day vitamin A in pregnancy and lactation and low vitamin A conditions (2.80 (2.53, 3.07) p < 0.0001). However, there was no association demonstrated at 14–16 weeks with 25,000 IU/day in obese and non-obese women and multiple sclerosis conditions (p = 0.19), respectively.

**Figure 4:**
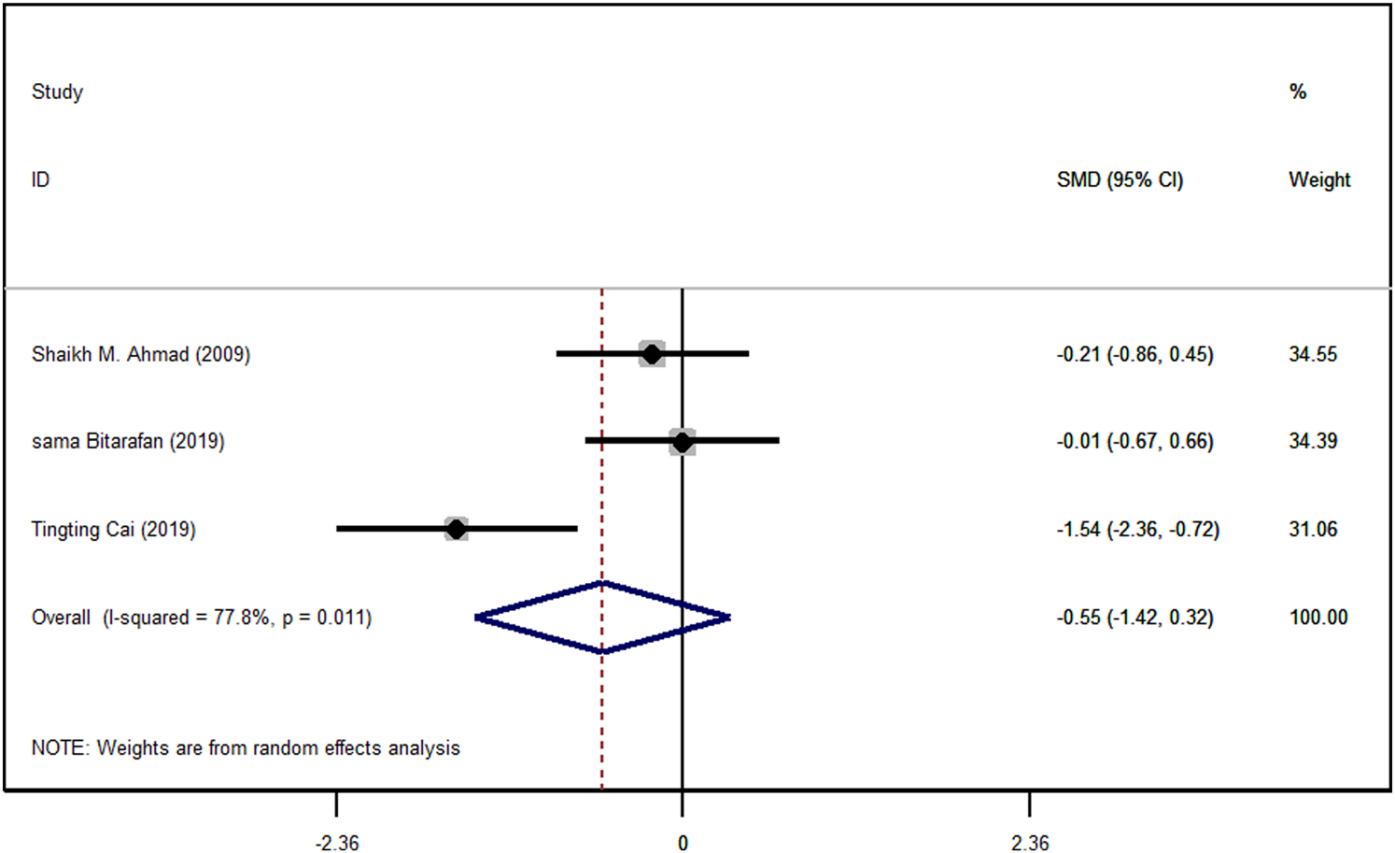
Forest plot for the effect of vitamin A supplementation on serum IL-6 concentrations, expressed as mean differences between intervention and control groups. Horizontal lines represent 95% CIs. *IL-6* interleukin 6, *SMD* standardized mean difference, *CI* confidence interval.

Regarding, three studies that included IL-6 in quantity, at first the forest plot analysis was performed in these studies. It’s shown that vitamin A supplementation has no significant association with IL-6 plasma concentration (p < 0.211) (Fig. 4). Subgroup analysis by dosage demonstrates a significant association between vitamin A supplementation and IL-6 in dosage with 50,000 with (p < 0.00001).

### The effects of vitamin supplementation on TNF-α and IL-6

Several studies for observing the association between vitamin A and TNF-α and IL-6 were reviewed. Long et al. showed that vitamin A is related to shorter E. coli fecal infections and lower IL-6 plasma levels^56^. Also, Tabone indicated that IL-6 was inversely associated with vitamin A plasma concentrations in patients with acute Plasmodium falciparum malaria^57^. Ekrem Boyali et al. showed in a before-and-after study on Taekwondo Players that vitamin A supplementation does not change IL-6 concentrations^58^ (Table 4).

**Table 4:** The effect of vitamin A on TNF-α and IL-6 in review studies.

Author, year	Country	Population (N)	Type of intervention	Age (year)	Sex	Dose (IU/day)	Duration (week)	Factor	Result
Kurt Z Long (2011)	Washington	127	Vit A	2	Both	32,500	40	TNF-α, IL-6	In supplemented children, detectable fecal TNF-α or IL-6 concentrations were associated with shorter E. coli infection durations
Kurt Z Long (2005)	Washington	127	Vit A	2	Both	32,200	60	IL-6	Vitamin A supplementation significantly decreased the IL-6 fecal level
M. D. TABONE (1992)	France	80	Vit A	36.7	Both	No reported	No reported	IL-6	IL-6 has inversely correlated with vitamin A supplementation
Mahdieh Abbasalizad Farhangi (2016)	Iran	56	Retinyl palmitate	4 months	Female	25,000 IU	16	IL-6	A significant decrease in IL-6 was observed at the end of the study (p < 0.05)
Sama Bitarafan (2019)	Iran	36	Retinyl palmitate	35	Both	25,000 IU	24	TNF-α IL-6	No significant difference between these two factors was found between the placebo and control groups
Shaikh M. Ahmad (2010)	Brazil	79	Retinol	12 months	Both	4 × 60 mg Equivalent	8	TNF-α IL-6	There was no significant difference in IL-6 but TNFα levels were decreased in the intervention group
Kristoffer Jarlov Jensen (2014)	Guinea-Bissau	311	Vit A	4.5 months	Both	50,000 IU	6	TNF-α IL-6	The results were not significant in the treatment and control groups
Ekrem Boyali (2017)	Turkey	10	Vit A	23.5	Male	100,000	4	IL-6 and TNF-α	Serum IL-6 levels in the athletes were not affected by either exercise or vitamin supplementation
Shaikh M. Ahmad (2009)	Bangladesh	36	Vit A	25	Male	240,000	56	IL-6	the high Vit A group tended to secrete less IL-6

Summary of clinical trials on the effects of vitamin A supplementation on inflammatory biomarkers (IL-6 and TNF-α). *IL-6* interleukin 6, *TNF-α* tumour necrosis factor-α. *M* male, *F* female, a Changes in cytokine concentrations are presented by common units for IL-6 (pg/mL) and TNF-α (pg/mL).

**Figure 5:**
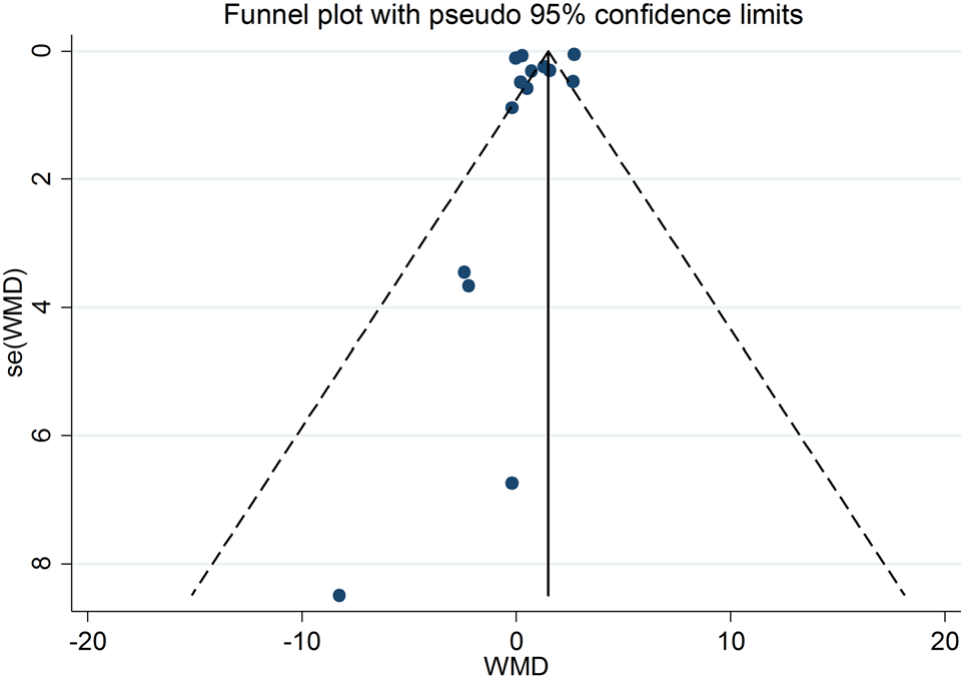
Funnel plot of CRP studies for finding publication bias. *CRP* C-reactive protein, *WMD* weighted mean differences.

The Egger and Begg analysis did not show any significant bias in studies reported (95% CI − 4.33, − 8.84, p value = 0.47). The funnel plot of studies did not highlight any significant publication bias (Fig. 5). Also, the risk of bias by RoB 2:0 for quality studies showed in each study appendix.

The Egger and Begg analysis did not show any significant bias in studies reported (95% CI − 500, 271, p value = 0.41). Also, the risk of bias RoB 2:0 for studies showed in each study in appendix.

However, the quality of evidence for IL-6 is poor due to substantial limits for inconsistency and imprecision. The examination of Grade assessment for CRP and TNF-revealed that there was intermediate quality of evidence due to serious limitations related to inconsistency (Table 5).

**Table 5:** GRADE profile of vitamin A supplementation for CRP, TNF-α, and IL-6.

Outcomes	Risk of bias	Inconsistency	Indirectness	Imprecision	Publication Bias	WMD (95% CI)	Quality of evidence
CRP	No serious limitation	Serious limitation^a^	No serious limitation	No serious limitation	No serious limitation	0.85 (0.30, 1.40)	⊕⊕⊕◯ moderate
TNF-α	No serious limitation	Serious limitation^a^	No serious limitation	No serious limitation	No serious limitation	− 5.17 (− 9.03, − 1.31)	⊕⊕⊕◯ moderate
IL-6	No serious limitation	Serious limitation^a^	No serious limitation	Serious limitation^b^	No serious limitation	− 0.55 (− 1.42, 0.32)	⊕⊕◯◯ Low

Grading of Recommendations Assessment, Development, and Evaluation (GRADE) of vitamin A supplementation for CRP, *CRP* C-reactive protein, *IL-6* IL-6 interleukin 6, *TNF-α* tumour necrosis factor-α. ^a^There is significant heterogeneity for CRP (I^2^ = 96.2%), TNF-α (I^2^ = 98.5%), and IL-6 (77.8%). ^b^There is no evidence of significant effects of vitamin A supplementation on IL-6 (confidence interval includes 0).

## Discussion

In this systematic meta-analysis included, we evaluated the association between vitamin A and inflammatory factors including CRP, TNF-α, and IL-6. we found vitamin A supplementation can exert a significant reducing effect on serum levels of TNF-α and IL-6, and also showed an increase in CRP concentration. We observed elevated plasma CRP levels as vitamin A dose increased to 25,000 IU/day. Particularly, low-dose vitamin A supplementation for a short duration elevated plasma CRP levels in a group of obese women, patients with pneumonia, multiple sclerosis^63^, and during infection conditions in children. Due to the large degree of variability across the CRP studies (96%), we first did a sensitivity analysis and discovered that the Ph Donnen et al. (2001) study that was conducted in children under infection settings was significant. We removed this study and performed the analysis without including this study. The heterogeneity reduces up to 0.85. However, we repeated this analysis for each studies. Finally, we found no evidence that excluding additional trials reduced heterogeneity. In addition, the baselines and standard deviation for the four investigations were not published, making it impossible to calculate increases in the CRP mean and changes in SD. We excluded this studies for calculating mean and SD changes for other studies. After that we performed a fixed effect analysis. We found result as following, there is positive correlation between vitamin A supplementation and CRP plasma concentration with SMD: 1.82, CI (1.71, 1.92), p value < 0.0001 and the heterogeneity reduces up to 20.2%. Although, CRP is not specific marker for assessing the inflammatory conditions and it is sensitive for many acute inflammations. We cannot consider the vitamin A as an inflammatory marker without considering other specific markers such as TNF-a and IL-6. Otherwise, the vitamin A plays many important roles as retinoic acid in gene expression that can active or suppress many cellular cascades^64^. Furthermore, vitamin A besides vitamin D showed important mechanisms for activation cellular signals^64,65^. Additionally, we observed a significant beneficial effect of vitamin A on TNF-α in studies that used retinyl palmitate at the dosage of (10,000 and 50,000 IU/day), during intervention for 15 and 44 weeks in pregnant and lactating women and people with hepatitis B. Surprisingly, high dose (240,000 IU/day) vitamin A supplementation for long-term resulted in a significant increase in serum TNF-α concentrations in individuals with lower levels of the vitamin. Compared to CRP, TNF-a is a sign of importance in inflammatory diseases. It activates a number of inflammatory indicators, such as adhesion molecules in endothelium damage, and performs significant roles in malignancies in addition to interleukin-1. It can also cause cachexia by influencing appetite^66–68^. These mechanisms involve altering the gene expression of NF-Kapa B^69^. Moreover, many studies have indicated that vitamin A supplementation and plasma concentrations have significant correlations with IL-6 plasma and fecal levels. Also, it decreases the IL-6 concentration based analysis in this study. Some studies did not find any association between vitamin A supplementation and the concentrations of IL-6 and TNF-α. It may be inferred that the most effective use of vitamin A for reducing inflammatory biomarker concentrations (TNF-α and IL-6) can be at high dosages for long durations. Albeit, vitamin A supplementation increases acute inflammatory markers such as CRP.

IL-6 is a pro-inflammatory cytokine which is increased in the early stage of infection^70^. When evaluating inflammatory disorders with Nutrice IV scores more than 400 ng, interleukin 6 is the best marker^71^. Additionally, it has the potential to evaluate cytokine storms in a variety of sepsis conditions. Additionally, numerous studies have shown that IL-6 is more reliable than other markers in determining the fatality rate in diseases caused by corona viruses^72^. However, some studies have reported the anti-inflammatory treatment for IL-6 by decreasing plasma TNF-α levels^73,74^. Although the plasma levels of IL-6 predict cardiovascular mortality, it has also been indicated that chronic IL-6 plasma predicts insulin resistance, obesity, and atherosclerosis^75,76^. Recently, a meta-analysis indicated that IL-6 is an important biomarker for diagnosing early-stage of coronavirus as well^77^. Rising plasma levels of TNF-α is associated with IL-6 and CRP in elderly people as well; TNF-α is correlated to atherosclerosis and Alzheimer’s in people over 100 years old^78^. It is considered an acute phase protein that correlates to inflammatory cytokines such as IL-6, CRP, and IL-8^79^.

Vitamin A deficiency increases inflammation, and insufficient intake of vitamin A increases chronic obstructive pulmonary disease risks (COPD). COPD has been correlated to chronic inflammation^63,80–82^. Also, many studies have reported that COPD increases certain cytokines such as IL-6, CRP, and TNF-α, which are the mediators of systemic inflammation^83,84^. Some studies have indicated that systemic inflammation is elevated in vitamin A deficiency^85,86^. The reason for this decreasing level of vitamin A in inflammatory conditions may be related to the low levels of retinol-binding protein (RBP) and the leakage of RBP to the extravascular space^26^. Moreover, some studies reported that plasma retinol decreases during inflammatory and septic conditions by secreting retinol in the urine^26,87,88^.

Several studies for observing the association between vitamin A and TNF-α and IL-6 were reviewed. Farhangi et al. (2016) performed a clinical trial study on 56 women in Iran. They supplemented 25,000 IU/day of vitamin A for 16 weeks. They found that vitamin A supplementation significantly decreases IL-6 status in intervention groups compared to placebo group^44^. Bitarafan et al. carried out a study in 2019 with a dosage of 25,000 IU/day for 24 weeks. They did not observe any difference in plasma IL-6 levels in the treatment group compared to the placebo group^89^. Ahmad performed a study in 2010, supplementing 240,000 IU/day retinol equivalent in children. They observed that retinol supplementation decreases TNF-α levels in intervention groups. In contrast, they did not observe any significant association between vitamin A supplementation and IL-6^90^. Ahmad^2^ showed that high vitamin A concentrations were related to low levels of IL-6. Jensen et al. carried out a study supplementing 250,000 and 50,000 IU/day vitamin A for 6 weeks in children. They did not find any significant association between vitamin A supplementation and plasma TNF-α or IL-6 levels^59^. Long et al.^55^ found that vitamin A supplementation in children significantly reduces fecal IL-6 in a treatment group compared to a placebo group. Also, Long et al. showed that vitamin A is related to shorter E. coli fecal infections and lower IL-6 plasma levels^56^. Tabone indicated in patients with acute Plasmodium falciparum malaria that IL-6 was inversely associated with vitamin A plasma concentrations^57^. Boyali et al. showed in a before-and-after study on Taekwondo Players that vitamin A supplementation does not change IL-6 concentrations^58^.

Given the foregoing, it appears that CRP, IL6, and TNF- responses to vitamin A varied depending on the dosage, length of intake, and health status of consumers. Supplementing with retinyl palmitate at doses of (10,000 and 50,000 IU/day) lowered TNF-α levels in pregnant and lactating women, as well as persons with hepatitis B., while in obese women, both retinyl palmitate and retinol supplementation elevated CRP levels, and unhealthy conditions such as MS and pneumonia were seen. More research is needed to see how vitamin A supplementation affects other inflammatory indicators in adults.

Mechanistic evidence for vitamin A's anti-inflammatory benefits is already being discovered. According to human and experimental research, vitamin A is necessary for proper immune system maintenance and function. In this regard. animal studies have shown vitamin A deficiency in animals had a lower antibody response than non-vitamin A deficiency^91^. Vitamin A also has anti-inflammatory properties. Vitamin A supplementation has been shown to improve a variety of inflammatory diseases^92^. In this context, a previous comprehensive study concluded that vitamin A deficiency causes inflammation and exacerbates pre-existing inflammatory conditions. Vitamin A supplementation, in particular, might help to reduce inflammation in some circumstances. Based on a previous study, vitamin A supplementation could increase protection against diverse pathogens^2^.

Numerous investigations have found that the antioxidant vitamin A has an effect, in part, by preventing the translocation of the transcription factor NFB and halting inflammatory cytokine release^93^. For example, animal research has revealed that vitamin A deficiency causes inflammation, fibrosis, increased collagen expression, and NF-B activation^92^.

In line with our study some authors have also suggested that, compared to long-term supplementation trials, several short- and midterm intervention studies have revealed health advantages for generally healthy and overweight persons, as evaluated by beneficial changes in inflammatory markers (IL-6, IL-8, TNF-α, IL-1b, CRP, NF-kB)^94^. Supplementation trials on individuals with chronic inflammation are more promising in current publications^29,95^. Furthermore, several researchers have indicated that carotenoid metabolites, such as enzymatic cleavage products (apocarotenals), are bioactive and serve as better targets for transcription factors like NF-kB and Nrf2^96,97^. Lower-to-intermediate concentrations may have anti-oxidant and pro-oxidant effects, while other increasing concentrations act pro-oxidatively^94^. The vitamin A-treated obese women's serum CRP concentrations significantly increased. Synthetic retinoids were shown to increase serum acute-phase proteins like CRP as a result^98^. It's been proposed that the rise is related to higher CRP synthesis in the liver or higher acute phase protein reactivity to key inducer cytokines like interleukin-6^98,99^.

The strength of the present systematic review and meta-analysis is that this is the first meta-analysis assessing associations between vitamin A and IL-6, TNF-α, and CRP. Subgroup analysis was performed in order to assess this relationship across different conditions. High-quality databases were searched, with restrictions for excluding related studies. The analysis was carried out on the plasma levels of these various inflammatory factors.

However, there are several limitations related to the present study. First, there were an inadequate number of studies at all ages for evaluating inflammatory factors such as CRP, IL-6 and TNF-α. Secondly, some studies performed were in cluster randomized trails and without placebo groups that excluded from analysis. Third, it was not possible to consider IL-6 analysis at the same level just three study included for IL-6, since some studies assessed fecal inflammatory markers, meaning the analysis could not be pooled. However, we did not register this review. Finally, there was considerable heterogeneity between the included studies. In the subgroup analysis, type and dosage of supplement could explain the variation between studies. Also, there is not adequate studies for assessing vitamin A and TNF-a and IL-6 for justification this relationship.

The result of this study demonstrates that supplementation of vitamin A at low and high dosages for short and long durations increases the CRP plasma concentrations Furthermore, vitamin A supplementation decreases the TNF-α concentrations. Therefore, there is an inverse association between vitamin A supplementation and plasma and fecal IL-6 concentrations in many infection conditions.

## Supplementary Information

Supplementary Information.

## Data Availability

This manuscript is a meta-analysis. All analysis was putted on manuscript and supplementary data.
