# Supplementary material for: Influence of Vitamin A supplementation on inflammatory biomarkers in adults: a systematic review and meta-analysis of randomized clinical trials
Source: Sci Rep. 2022 Dec 10;12:21384. doi: 10.1038/s41598-022-23919-x (PMC9735279; doi:10.1038/s41598-022-23919-x)
Supplement: Supplementary file 1 — Supplementary Information. [file 41598_2022_23919_MOESM1_ESM.docx]

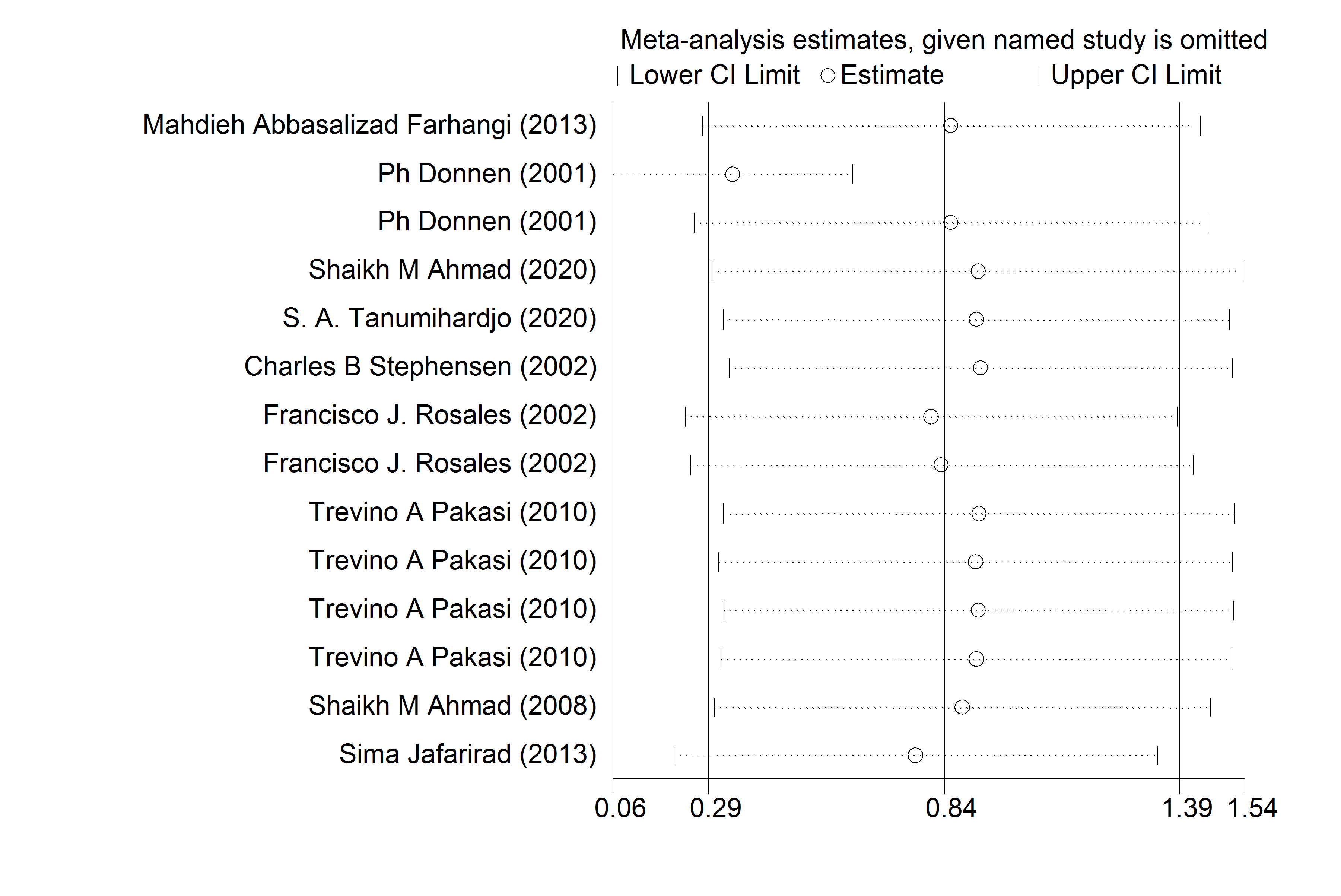
Appendix 1. Sensitive analysis for funding weight of studies and effect of each study in result separately


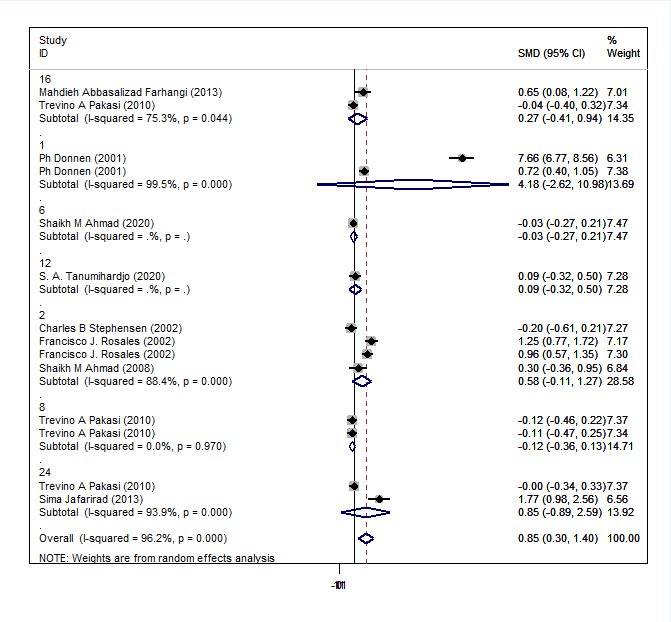


Appendix 2. Forest plot of association of the vitamin A supplementation in subgroups by duration (weekly).


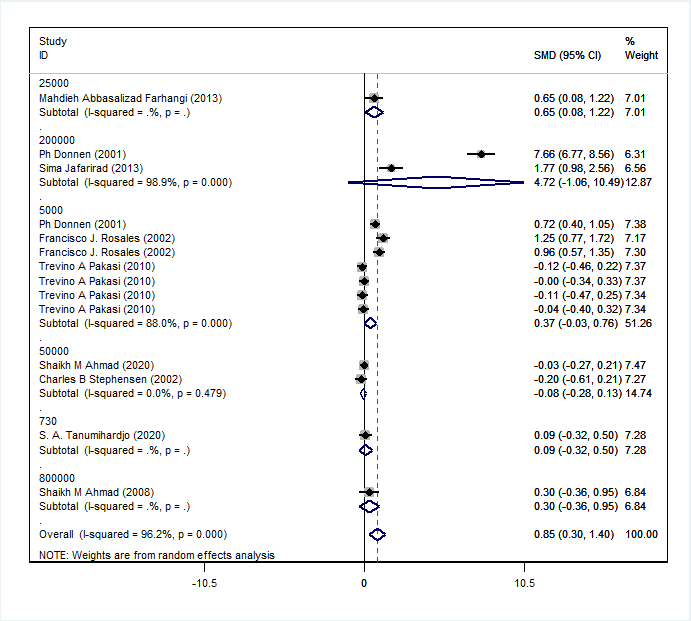


Appendix 3. Forest plot of association of the vitamin A supplementation in subgroups by dosage (IU/d)


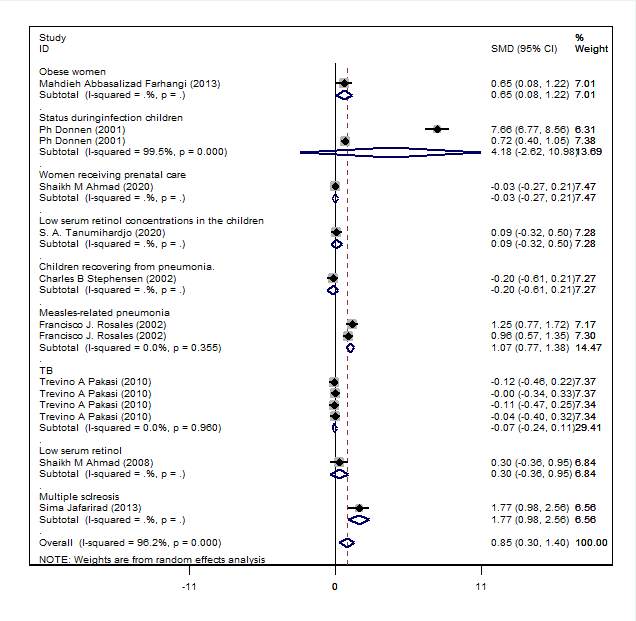


Appendix 4. Forest plot of association of the vitamin A supplementation in subgroups by conditions.


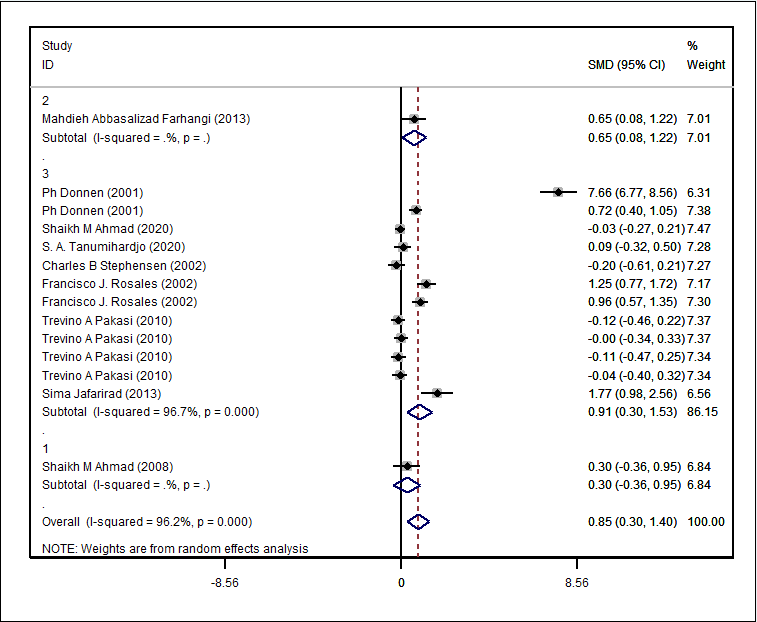


Appendix 5. forest plot of association of the vitamin A supplementation in subgroups by sex. men; 1, women: 2 and both;3.


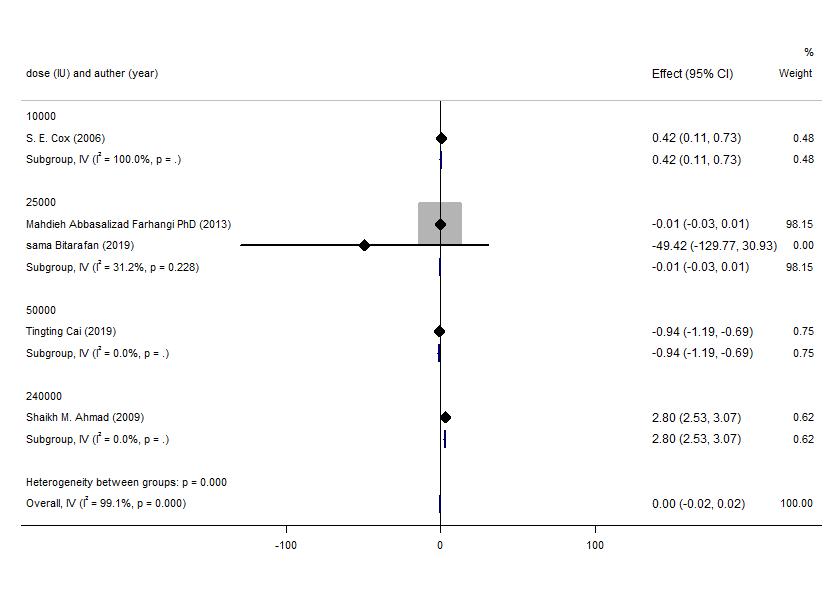


Appendix 6. The association between vitamin A supplementation and TNF-a subgroup by dosage.


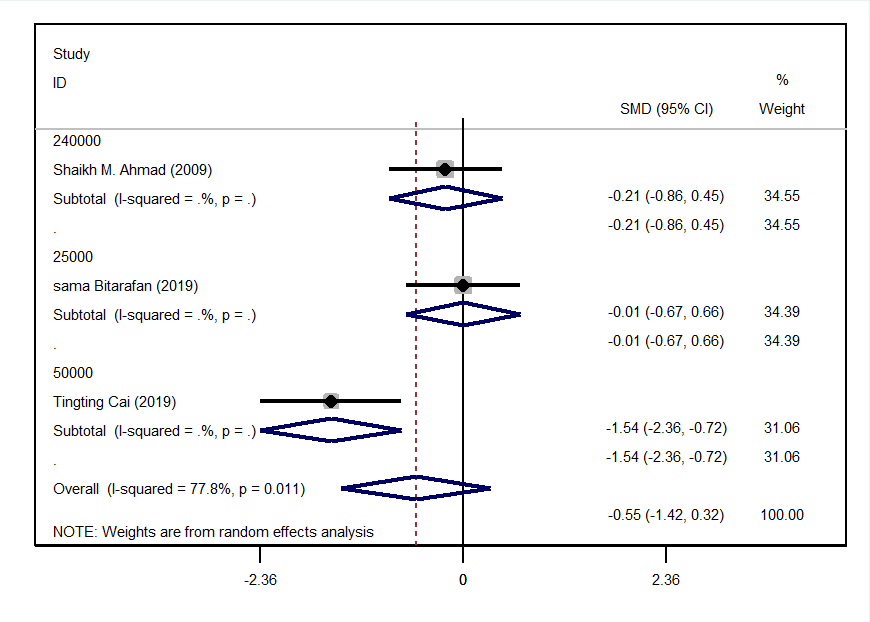


Appendix 7. The association between vitamin A supplementation and IL-6 subgroup by dose.


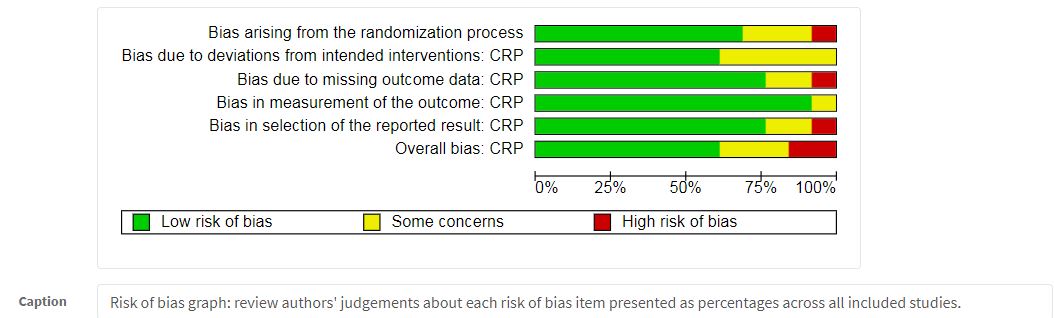


Appendix 8. Assessing publication bias of studies included for CRP by RoB 2. (in summary)


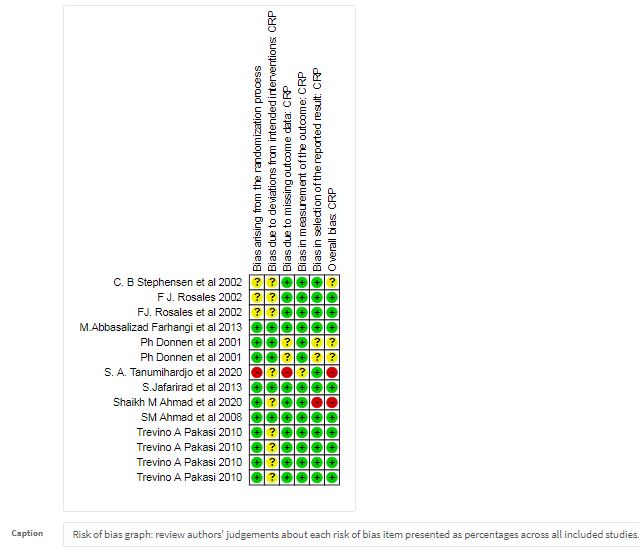


Appendix 9. Assessing publication bias of studies included for CRP by RoB 2. (for each study)


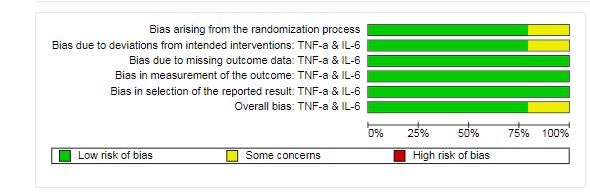


Appendix 10. Assessing publication bias of studies included for TNF-a and IL-6 by RoB 2. (in summary)


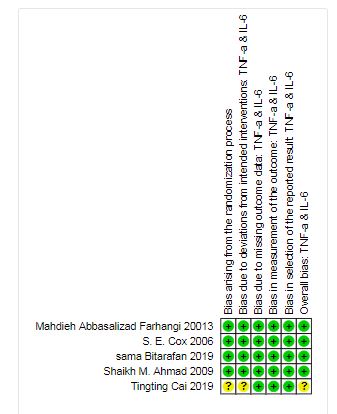


Appendix 11. Assessing publication bias of studies included for TNF-a and IL-6 by RoB 2 (based on each study)


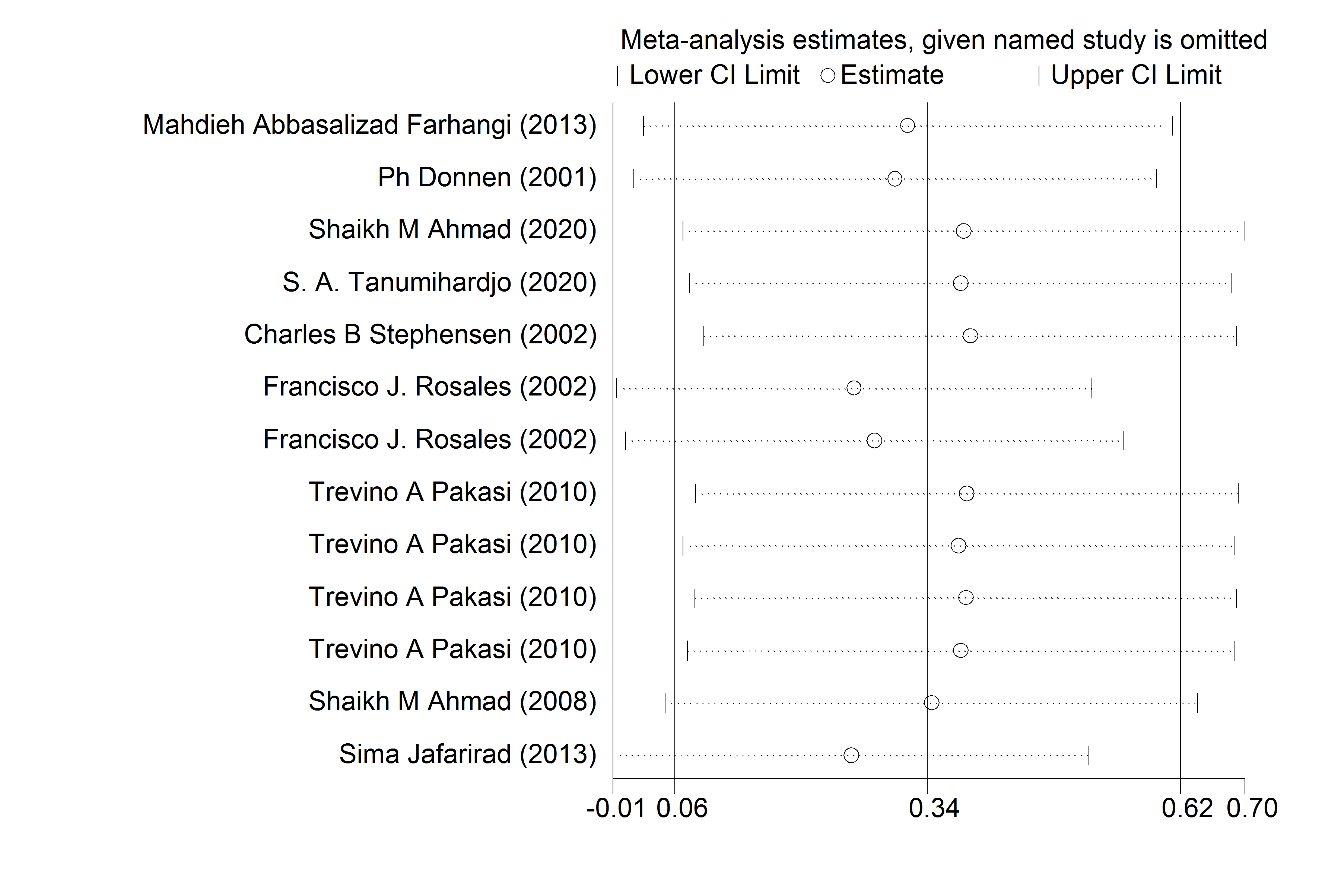


Appendix 12. sensitivity analysis after removing the study with a high weight effect .


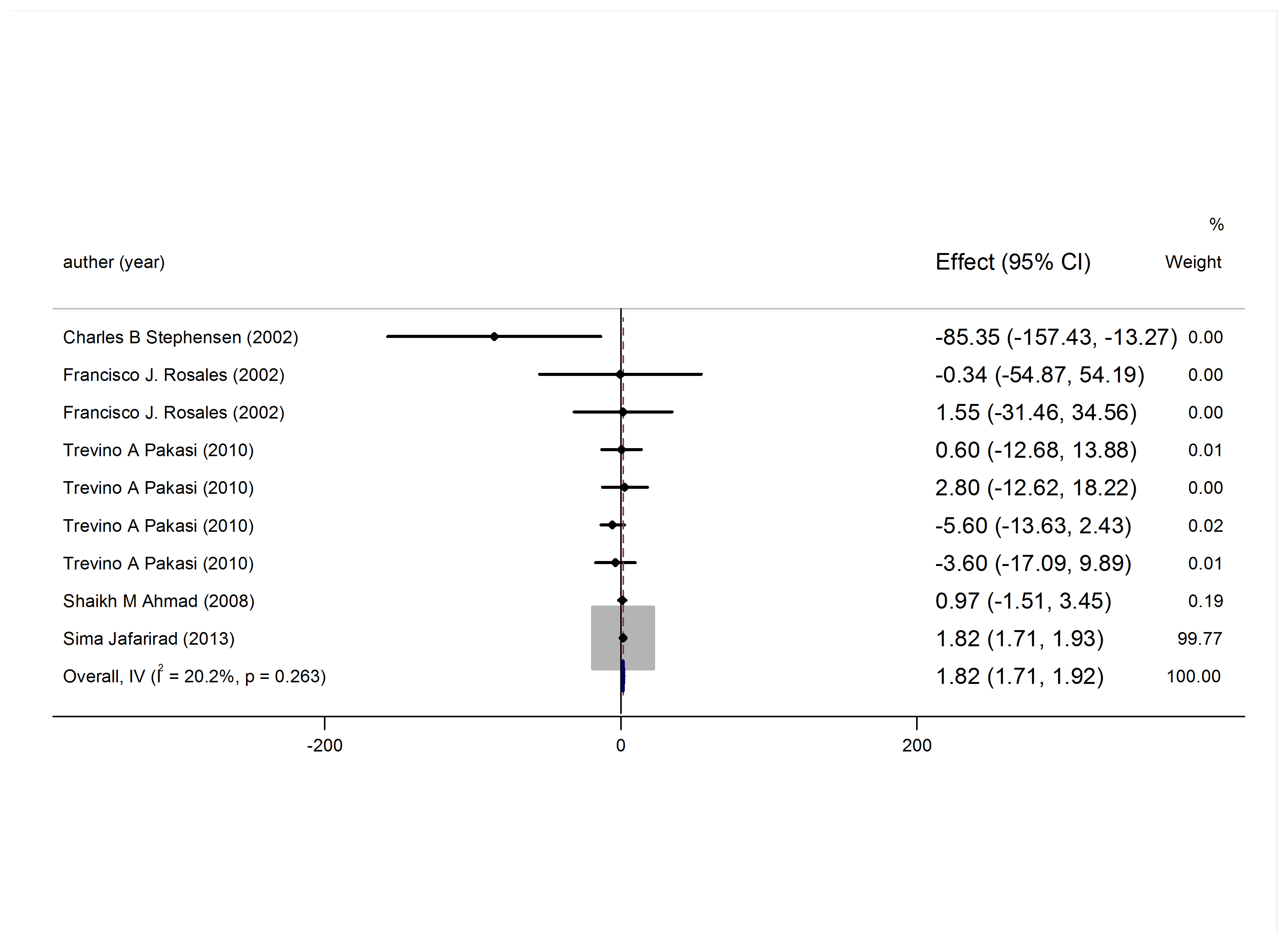


Appendix 13. Forest plot the effect of vitamin A supplementation and CRP concentration (after removing the studies that they were not reported changes)
